# Postoperative wound care protocol prevents surgical site infection after craniotomy

**DOI:** 10.1017/ice.2024.134

**Published:** 2024-12

**Authors:** Mariya Kovryga Kornick, Eunjung Lee, Lisa Wilhelm, Janice White, Oh-Hyun Cho, Michelle Paff, Frank P.K. Hsu, Jefferson Chen, Linda Dickey, Susan S. Huang

**Affiliations:** 1 Department of Epidemiology and Infection Prevention, University of California, Irvine Health, Orange, USA; 2 Division of Infectious Diseases, University of California, Irvine School of Medicine, Irvine, USA; 3 Division of Infectious Diseases, Department of Internal Medicine, Soonchunhyang University Seoul Hospital, Soonchunhyang University College of Medicine, Seoul, Republic of Korea; 4 Department of Nursing Education, University of California, Irvine Health, Orange, USA; 5 Nursing Administration, University of California, Irvine Health, Orange, USA; 6 Division of Infectious Diseases, Soonchunhyang University Cheonan Hospital, Cheonan, Republic of Korea; 7 Department of Neurological Surgery, University of California, Irvine School of Medicine, Orange, USA

## Abstract

**Background:**

Postoperative wound care after craniotomy is not standardized.

**Objective:**

Evaluate the impact of a standardized post-craniotomy wound care protocol on surgical site infection (SSI).

**Design and Setting:**

Prospective quasi-experimental single-center intervention cohort study involving adult patients undergoing craniotomy at a 461-bed academic medical center in Orange County, California from January 2019–March 2023 (intervention) compared to January 2017–December 2018 (baseline).

**Methods:**

A postoperative neurosurgical wound care protocol was developed involving chlorhexidine cloths to remove incisional clots and to clean the surgical incision and adjacent hair after craniotomy surgery. Protocol adherence was monitored by routine inpatient surveillance of wounds and photo-documentation for real-time feedback to surgeons and nursing staff. Impact of the intervention was assessed using multivariable regression models.

**Results:**

There were 3560 craniotomy surgeries and 62 (1.7%) SSIs; 1251 surgeries and 30 (2.4%) SSIs during baseline, and 2309 surgeries and 32 (1.4%) SSIs during intervention. Process evaluation after implementation found significant decreases in incisional clots, erythema, drainage, and unclean hair. In multivariable analysis, the intervention was associated with fewer SSI (odds ratio (OR): 0.5 (0.3, 0.9), *P* = 0.02).

**Conclusions:**

A standardized post-craniotomy wound care protocol involving cleaning of the incision and adjacent hair, including removal of incisional clots with chlorhexidine cloths was effective in reducing the risk of SSI.

## Introduction

Nearly 50,000 craniotomy procedures are performed annually in the United States.^1^ Surgical site infections (SSIs) after craniotomy cause substantial morbidity and mortality, often requiring extended hospital stays and additional operations.^2–4^

Approximately 70% to 95% of SSIs are caused by endogenous flora colonizing the head, neck, and shoulder area.^5^ Most SSI prevention strategies involve preoperative chlorhexidine (CHG) bathing, incisional skin antisepsis, appropriate hair removal, antibiotic prophylaxis, intraoperative vancomycin powder and irrigation solution, various closure methods and/or materials, and managing postoperative hyperglycemia.^6–15^ Some also include screening for and decolonizing *Staphylococcus aureus* carriers.^16^

In contrast to preoperative and perioperative SSI prevention guidance,^17^ postoperative craniotomy SSI prevention efforts have not been standardized. Evidence-based guidelines do not recommend a specific postoperative wound care protocol.^18^ Although various publications for postoperative wound management after craniotomy have included options for advanced dressings^13^ and prophylactic topical antibiotic application to the incision,^19^ studies focusing on care for postoperative incisional wounds and adjacent hair are lacking.^6^

Post-craniotomy incisional care is complicated by local wound factors, including the high vascularity of the face and scalp (which contributes to clot formation),^20^ the high microbial colonization of the sebaceous scalp and head,^5^ and the presence of hair, as some neurosurgeons prefer to minimize hair clipping along the incision line.^21^ Hair not only causes physical contamination of the incision, but it raises questions about how to keep the area clean and keep adhesive dressings in place.

The objectives of this study were to design an effective postoperative wound care protocol for cleaning craniotomy incisions as well as the surrounding hair, and to evaluate the impact on SSI.

## Methods

We conducted a prospective quasi-experimental intervention cohort study of patients undergoing craniotomy at the University of California Irvine Medical Center, a 461-bed academic medical center. We included a 24-month baseline period (January 2017–December 2018) and a 48-month intervention period (January 2019–March 2023), which excluded a 3-month period from October to December 2020 when the intervention program was halted due to the first COVID-19 pandemic surge in California. The program restarted in January 2021, following the December 2020 rollout of the first COVID-19 vaccine to healthcare providers. This quality improvement project was conducted for hospital operations and was exempt from review by an institutional review board.

Descriptive data on craniotomy surgeries were collected from the electronic health record and included common characteristics required for national reporting. This included patient demographics, body mass index, diabetes status, surgical urgency (elective versus emergent), and surgical wound class.

### Development of a post-craniotomy wound care protocol

A multidisciplinary performance improvement team was convened at the end of 2018 to create a standardized post-craniotomy wound care protocol to address dressing integrity, incisional clots, postoperative hair cleanliness, and wound care. The team included wound care nurses, neurosurgical nurse specialists, neurosurgeons, and infection prevention leaders. At the time, monthly SSI rates were commonly 2%–4% with most pathogens representing skin flora.

Multiple products were trialed for removing incisional clots: hydrogen peroxide (0.3%, 1.5%, 3%), 2% CHG sponge and swab stick applicators, and 2% CHG cloths. We applied products two-at-a-time on opposite ends of a convenience sample of incisional clots to test their effect on clot removal. We began with lower hydrogen peroxide percentages to find the minimum effective concentration since higher concentrations may cause skin cell damage and adversely affect wound healing.^22^ Nevertheless, none of the tested dilutions were effective for clot removal. In trialing CHG, 2% CHG sponge and swab applicators were impractical because their surface material became trapped by metal staples. Warmed 2% CHG cloths were consistently able to soften, loosen, and remove dried blood clots. The developed protocol involved squeezing 2% CHG solution from a warmed impregnated cloth onto the incision and laying the cloth flat for 15 minutes to breakdown dried blood/clots before cleaning the incision with a new CHG cloth.

To address unkempt and/or greasy hair over the incision, the two inches of peri-incisional skin and hair were included in the existing hospital-wide daily CHG bed bathing protocol. This protocol already included cleaning the proximal 6 inches of any surgical drain.^23^ To keep hair away from the incision, soft baby hair ties were used, avoiding hard clips or braiding to prevent abrasion or pressure injury. If a dressing was present, cleaning occurred over and around the dressing. This protocol was adopted once the surgical dressing was removed or on postoperative day 1, whichever came first. Dressings, if used, were typically removed on postoperative day 2, with variation from 1–3 days among neurosurgeons. In addition, shampooing began on postoperative day 3 and occurred every 3 days using 4% rinse-off CHG soap for long hair and 2% CHG cloths for short hair or bald heads. No-rinse shampoo caps were eliminated. The final developed inpatient protocol is found in Supplemental Appendix 1.

### Intervention implementation

The intervention period included education and protocol monitoring of all postoperative craniotomy wounds twice-weekly with photo-monitoring by a rounding team consisting of a neurosurgery clinical specialist and an infection preventionist. Adoption of the protocol was facilitated by dissemination of practices via clinical updates, presentations, and demonstrations at the nursing practice councils and department meetings. Protocol ambassadors among doctors and nurses were engaged to promote the protocol among their peers.

Protocol lapses were addressed with just-in-time educational feedback to bedside nurses, and photos of protocol lapses were shared by email with the attending surgeon and unit nurse manager to ensure appropriate response consistent with the protocol. Preoperative processes were unchanged and included preoperative CHG bathing and hair washing for inpatients awaiting surgery.

### Evaluation of the impact of the post-craniotomy wound care protocol on surgical incision appearance

Immediately before and after the protocol was implemented, we conducted a series of point prevalence assessments of post-craniotomy incisions, evaluating hair cleanliness, and presence of incisional clots, drainage, edema, erythema, and dehiscence of postoperative wounds. Assessments involved visual assessments and twice-weekly photo-surveys of all inpatients undergoing craniotomy during the end of the baseline period (October–December 2018) and shortly after intervention launch (March–August 2019). Data on incisional appearance were assessed by calculating and comparing the percent of problematic elements among all assessments in the baseline and intervention periods using chi-square tests.

### Evaluation of the impact of the post-craniotomy wound care protocol on craniotomy SSI

SSIs were identified according to the Centers for Disease Control and Prevention’s National Healthcare Safety Network criteria, and described by depth (superficial, deep incisional, and organ space). The proportion of SSI among craniotomy surgeries was calculated for baseline and intervention periods. Bivariate analyses were conducted by entering patient and surgical characteristics, including period, as independent variables one-by-one into generalized-linear mixed models for the outcome of SSI. Models accounted for clustering by surgeon. Multivariable models were conducted with independent variables entered based upon clinical importance. Significance was assessed using a two-tailed alpha of 0.05 and conducted using SAS version 9.4 (SAS Institute, Cary, NC).

## Results

A total of 3560 craniotomies were performed across the study period, including 1251 during the baseline period and 2309 during the intervention period. Patient characteristics were similar across the baseline and intervention periods, although patients in the intervention period were slightly more likely to have a body mass index of 30 or greater, diabetes, and an emergent operation or surgery due to trauma (Table 1).

**Table 1.** Characteristics and surgical descriptors of patients undergoing craniotomy

Characteristics	Overall N (%)	Baseline (24-mo) N (%)	Intervention (48-mo) N (%)
N	3560	1251	2309
Age in years (median (IQR))	55 (40, 67)	54 (39, 66)	56 (40, 67)
Male	2022 (56.8)	692 (55.3)	1330 (57.6)
Diabetes	708 (19.9)	214 (17.1)	494 (21.4)
Body mass index ≥30	934 (26.2)	293 (23.4)	641 (27.8)
Prior craniotomy in past year	567 (15.9)	208 (16.6)	359 (15.5)
Wound Class			
I	2945 (82.7)	1022 (81.7)	1922 (83.2)
II	422 (11.9)	157 (12.5)	265 (11.5)
III	72 (2.0)	23 (1.8)	49 (2.1)
IV	121 (3.4)	49 (3.9)	73 (3.2)
Trauma	244 (6.9)	54 (4.3)	190 (8.2)
Emergent operation	663 (18.6)	177 (14.1)	486 (21.0)
Endoscopic	317 (8.9)	113 (9.0)	204 (8.8)
Posterior fossa surgery	193 (5.4)	49 (3.9)	144 (6.2)
Procedure duration in hours (median (IQR))	2.6 (1.7, 3.8)	2.4 (1.5, 3.6)	2.7 (1.8, 4.0)

Note. IQR, interquartile range.

### Intervention impact on craniotomy incision appearance

**Figure 1.**
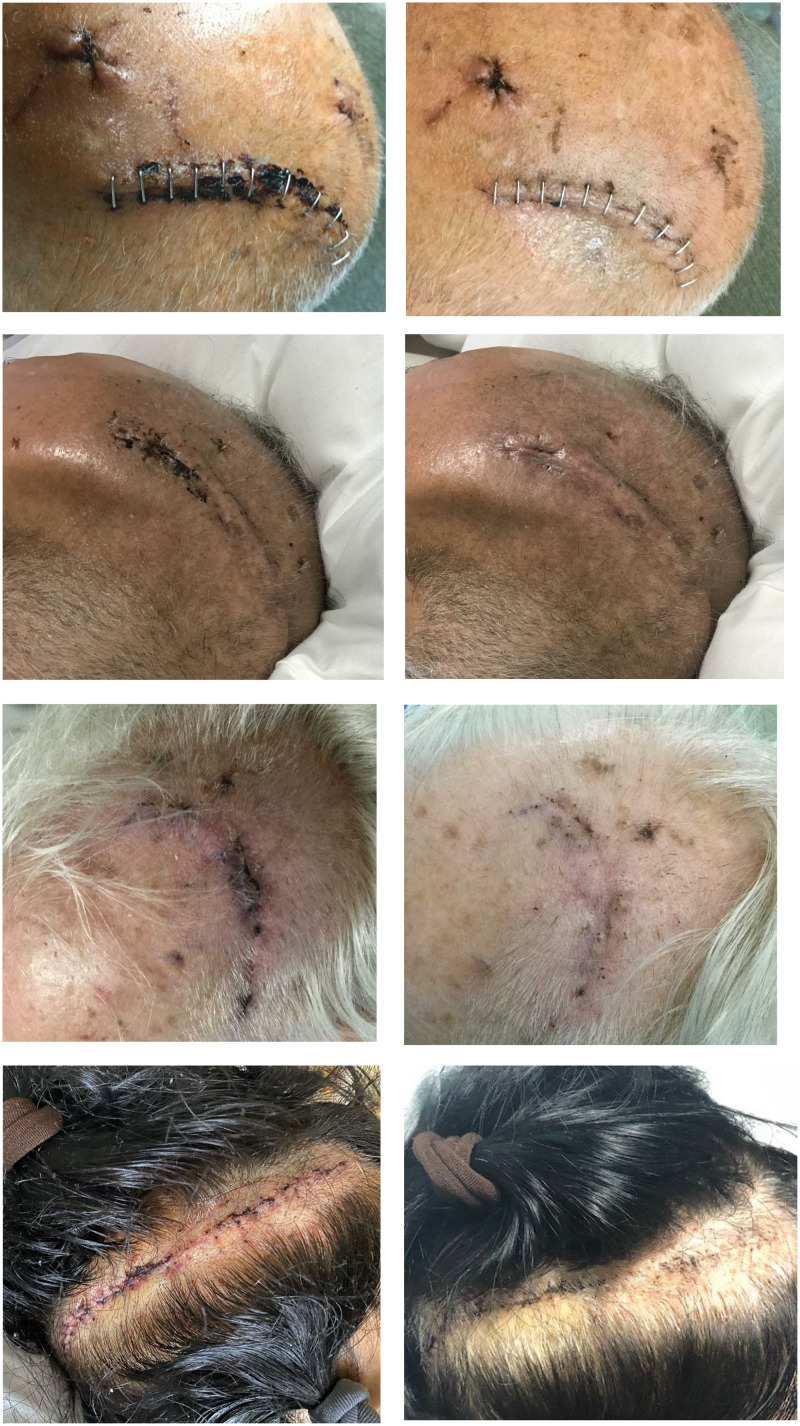
Examples of pre-intervention and post-intervention craniotomy wounds during photo-documentation monitoring.

The pre- and post-evaluation of craniotomy incision appearance involved 308 assessments of 124 patients, including 101 assessments of 45 patients (2.2 photos per incision) during the pre-intervention assessment period, and 207 assessments of 79 patients (2.6 photos per incision) during the assessment period shortly after the intervention began. Characteristics of patients involved in these visual assessments of incisions and results from the visual assessments are shown in Table 2. The intervention was associated with a reduction in problematic wound issues, including a 46.3% reduction in incisional clots (*P* < 0.001), 65.0% reduction in erythema (*P* < 0.001), 68.2% reduction in greasy hair (*P* < 0.001), and 90.0% reduction in drainage (*P* = 0.02). Pictures of wounds before and after completing the wound care protocol are shown in Figure 1.

**Table 2.** Visual assessments of craniotomy incisions pre- and post-intervention, patient characteristics and findings

	Pre-Intervention (3-mo) N (%)	Post-Intervention (6-mo) N (%)
**Characteristics of Assessed Patients**		
N	45	79
Age in years (median (IQR))	60 (53-69)	57 (43-66)
Male	23 (51.1)	51 (64.6)
Diabetes	11 (24.4)	21 (26.6)
Body mass index ≥ 30	8 (17.8)	17 (21.5)
Prior craniotomy in past year	6 (13.3)	5 (6.3)
Reason for surgery		
Brain tumor	16 (35.6)	29 (36.7)
Hemorrhage (trauma)	15 (33.3)	19 (24.0)
Hemorrhage (non-trauma)	12 (26.7)	21 (26.6)
Other	2 (4.4)	10 (12.7)
Emergent operation	26 (57.8)	38 (48.1)
Wound Class I	42 (93.3)	77 (97.5)
Appropriate antibiotic prophylaxis	43 (95.6)	78 (98.7)
Preoperative CHG bathing^a^	18 (94.7)	35 (85.4)

Visual Assessment Findings			
	Pre-Intervention (3-months) N (%)	Post-Intervention (6-months) N (%)	P-Value
Number of assessments	101	207	
Blood clots			
Any	70 (69.3)	77 (37.2)	**< 0.001**
< 50%	36 (35.6)	66 (31.9)	0.52
≥ 50%	34 (33.7)	11 (5.3)	**< 0.001**
Redness	28 (27.7)	20 (9.7)	**< 0.001**
Edema	14 (13.9)	21 (10.1)	0.34
Drainage	5 (5.0)	1 (0.5)	**0.02**
Dehiscence	2 (2.0)	0 (0.0)	0.11
Greasy hair^b^	46 (76.7)	30 (24.4)	**< 0.001**
Suture (vs staple)	33 (32.7)	68 (32.9)	1.00

Note. IQR, interquartile range; CHG, chlorhexidine.

a Evaluated among elective surgeries: N = 19 pre-intervention, N = 41 post-intervention.

b Patients with hair evaluated.

### Intervention impact on craniotomy surgical site infections

**Figure 2.**
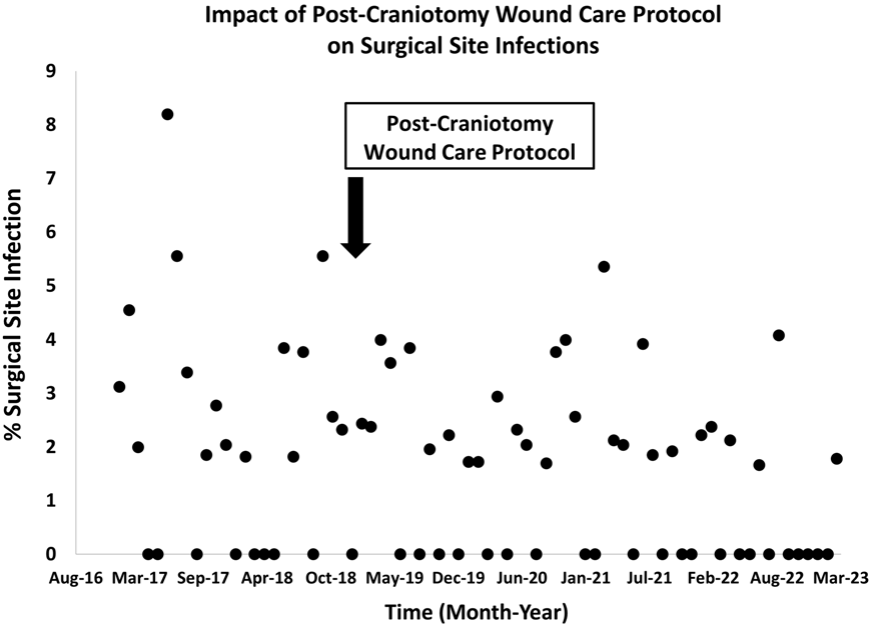
Scatterplot of monthly craniotomy surgical site infection rates before and after introduction of the post-craniotomy wound care protocol demonstrating reduction in monthly rates after implementing soft hair ties to keep hair away from the incision, warmed 2% chlorhexidine (CHG) cloths for cleansing and removal of incisional clots on a daily basis, and CHG shampoo for hair every 3 days.

Across the entire study period, among the 3560 craniotomy surgeries, there were 62 SSIs. During the 24-month baseline period, there were 30 (2.4%) SSIs out of 1251 surgeries. This included 2 (6.7%) superficial, 3 (10.0%) deep incisional, and 25 (83.3%) organ-space infections. During the 48-month intervention period, there were 32 (1.4%) SSIs out of 2309 surgeries, including 2 (6.3%) superficial, 3 (9.4%) deep incisional, and 27 (84.4%) organ-space infections. Figure 2 displays the percent of SSI among craniotomies performed each month before and after the introduction of the post-craniotomy wound care protocol.

Types of pathogens associated with SSI are found in Supplemental Appendix 2.

In bivariate analyses, the intervention period (odds ratio (OR) = 0.6 (0.3, 1.0), *P* = 0.03) and diabetes (OR = 0.4 (0.2, 1.0), *P* = 0.05) were associated with a decreased odds of SSI at alpha <0.1 while procedure duration (per hour) (OR = 1.2 (1.1, 1.3), *P* < 0.001), previous craniotomy within the past year (OR = 1.8 (1.0, 3.2), *P* = 0.06), and emergent or trauma-related surgery (OR = 1.7 (0.9, 3.0), *p* = 0.08) were associated with increased odds of SSI. In multivariable analysis, the wound care intervention remained significantly associated with reduced SSI while procedure duration, and emergent or trauma-related surgery remained positively associated with SSI (Table 3).

**Table 3.** Multivariable analysis of factors associated with post-craniotomy surgical site infections

Variable	Odds Ratio (95% CI)	*P* **-Value**
Wound Care Protocol Intervention (vs Baseline)	0.5 (0.3, 0.9)	**0.02**
Age (referent <40 years)		0.61
40–49	1.0 (0.4, 2.2)	
50–59	1.2 (0.6, 2.4)	
60–69	0.8 (0.4, 1.9)	
70–79	0.4 (0.1, 1.5)	
80+	1.5 (0.5, 4.6)	
Male sex	1.2 (0.7, 2.1)	0.48
Diabetes	0.4 (0.2, 1.0)	0.06
Body mass index ≥30	1.3 (0.7, 2.3)	0.42
ASA score (referent ≤2)		0.59
3	1.7 (0.6, 4.3)	
4+	1.6 (0.5, 4.9)	
Prior craniotomy in past year	1.8 (1.0, 3.3)	0.07
Trauma or emergency surgery	2.1 (1.0, 4.3)	**0.05**
Dirty or contaminated wound class	1.0 (0.4, 2.9)	0.99
Procedure duration in hours	1.2 (1.1, 1.3)	**<0.001**
Endoscope used	0.6 (0.2, 2.1)	0.47
Posterior fossa surgery	0.5 (0.1, 2.3)	0.40

Note. ASA, American Society of Anesthesiologists.

## Discussion

We found that a post-craniotomy wound care protocol involving warmed CHG-impregnated cloths to remove incisional clots as well as to clean the incision and adjacent hair, in conjunction with soft ties to keep hair away from the incision, was associated with a 50% lower odds of SSI. This highlights the potential value of scalp wound care protocols for postoperative craniotomy care.

Primarily closed surgical wounds begin to seal within 24-48 hours postoperatively. For this reason, we encouraged cleansing of the incision and surrounding hair starting on postoperative day 1. The American College of Surgeons and Surgical Infection Society’s SSI guidelines state that early showering does not increase SSI and can be encouraged at the surgeon’s discretion.^18^ These data suggest that early postoperative wound care prevents SSI by reducing bacteria, sweat, and dirt accumulation near the incision.^24^ This may be particularly important for the scalp, given commensal bacteria associated with hair follicles and sebaceous glands. Furthermore, postoperative hair washing may be more important when the hair is minimally clipped, as is the preference of several surgeons at our institution.

Before the intervention, we identified several challenges to effective cranial wound cleaning, including inconsistent wound care, and variation in cleansing products, dressings, timing of dressing removal, and instructions about hair washing. There were unclear roles and responsibilities between neurosurgery and nursing for incisional care. This inconsistency likely led to the pre-intervention findings of unkempt and greasy hair and variable dressing practices.

In addition, the scalp’s vascularity often leads to incisional clots, a nutrient source for scalp and facial bacteria. There was reluctance to clean the incision until it was demonstrated that CHG cloths could remove clots without disrupting the wound or impeding healing.

The post-craniotomy wound care protocol appeared to reduce all depths of SSI. The odds of SSI were reduced by 50% while the distribution of superficial, deep, and organ-space SSIs was unchanged. This suggests that a substantial proportion of post-craniotomy SSI derives from scalp bacteria that enter the incision and cause SSI of all depths, particularly given the proximity of skin to cranial bone.

We highlight the value of a multidisciplinary approach to reduce craniotomy-associated SSI. Engagement of neurosurgeons, wound care nurses, nursing leadership, and infection prevention enabled the formation of a consensus protocol, provided support for training and education, and allowed twice-weekly protocol adherence monitoring of all postoperative craniotomy wounds with rapid feedback to clinical partners and frontline staff for lapses, which were quickly rectified. Photos of the wounds were particularly impactful as an effective visual tool for providing feedback on practice improvement opportunities and increasing adherence to the protocol.

While previous research has focused on multiple preoperative and perioperative practices to reduce SSIs, these results showed that a postoperative antiseptic wound care protocol could reduce nearly half of the SSIs at an academic medical center. Limitations include the single-center nature of study, and the fact that the intervention was interrupted by the COVID-19 pandemic. Nevertheless, the fact that the intervention was only halted for 3 months reflected the adoptability of the protocol and the value conferred by patient care staff.

In conclusion, the implementation of a standardized post-craniotomy wound care protocol using soft hair ties to keep hair away from the incision, warmed 2% CHG cloths for cleansing and removal of incisional clots on a daily basis, and CHG shampoo for hair every 3 days led to visible improvements in incisional and peri-incisional hygiene, as well as marked reduction in SSIs when paired with ongoing photo-documentation and feedback for protocol adherence.

## Supporting information

Kovryga Kornick et al. supplementary material 1 Kovryga Kornick et al. supplementary material

Kovryga Kornick et al. supplementary material 2 Kovryga Kornick et al. supplementary material

## Supplementary material

The supplementary material for this article can be found at https://doi.org/10.1017/ice.2024.134
