# Supplementary material for: Postoperative wound care protocol prevents surgical site infection after craniotomy
Source: Infect Control Hosp Epidemiol. 2024 Oct 14;45(12):1399–404. doi: 10.1017/ice.2024.134 (PMC11663463; doi:10.1017/ice.2024.134)
Supplement: Kovryga Kornick et al. supplementary material 1 — Kovryga Kornick et al. supplementary material [file S0899823X2400134Xsup001.docx]

Supplemental Appendix 2. Types of pathogens associated with SSI.

| SSI Associated Microorganisms | Baseline  (24 months)  N (%) | Intervention  (48 months)  N (%) |
| --- | --- | --- |
| Culture positive | 30 | 26 |
| Single pathogen | 21 (70%) | 15 (57.7%) |
| - Gram-positive organism | 16 (76.2%) | 12 (80%) |
| - - Skin commensal | 15 (93.7%) | 10 (83.3%) |
| - - Noncommensal | 1 (6.3%) | 2 (16.7%) |
| - Gram-negative organism | 5 (23.8%) | 3 (20%) |
| Polymicrobial | 9 (30%) | 9 (34.6%) |
| Candida species | 0 (0%) | 2 (7.7%) |
