# Supplementary material for: Postoperative wound care protocol prevents surgical site infection after craniotomy
Source: Infect Control Hosp Epidemiol. 2024 Oct 14;45(12):1399–404. doi: 10.1017/ice.2024.134 (PMC11663463; doi:10.1017/ice.2024.134)
Supplement: Kovryga Kornick et al. supplementary material 2 — Kovryga Kornick et al. supplementary material [file S0899823X2400134Xsup002.docx]

| **Post-Craniotomy Surgical Wound Care Protocol** | | | | |
| --- | --- | --- | --- | --- |
| **Daily Till Discharge** | 1. Use elastic bands/hair ties to keep hair away from wound. 2. With warmed 2% chlorhexidine (CHG) cloths:  - Clean the incision and surrounding **2 inches** of skin and hair (use new wipe for the incision area/wound). A CHG applicator may be used as an alternative. - If a surgical dressing is present, clean with a CHG cloth over non-gauze dressings, and clean the skin and hair around the dressing. Clean the incision once dressing removed. - If NO dressing is used, wound cleaning may begin Post-Op Day (POD) 1. - Hair/skin around the dressing should be cleaned. - Clean front of the neck and face (avoid eyes and ear canal). - Clean back of neck to the hairline and behind ears. - **Remove dried blood/clots** with warm CHG cloth. Drip solution on incision and lay cloth for 15 minutes to breakdown clots. Clean with new cloth. - If oozing occurs, apply pressure until stops. If wound continues to ooze despite pressure, notify Neurosurgery.  1. If drain(s) present:  - After cleaning incision, clean proximal **6 inches** of drain and surrounding  **2 inches** of skin and hair with new 2% CHG cloth. - Hold drain tube while cleaning and do not pull. | | | |
| **POD 3 Till Discharge** | 1. **If no drain is present, clean peri-incisional hair daily per above protocol, plus:**   **Start shampooing on day 3 and every 3 days** until discharge.   - Wash hair with 4% CHG soap and rinse with water; do not use shampoo caps. - **CHG-allergic patients:** Wash hair with baby shampoo, do not use shampoo caps. - Inform the clinical team when the next shampoo is due: write date on patient room whiteboard, add reminder on nursing task list.  1. **If drain is present, clean peri-incisional hair daily per above protocol, plus: Use CHG cloths to clean the head and all hair.** | | | |
| **Products** | **4% CHG Hair Soap**  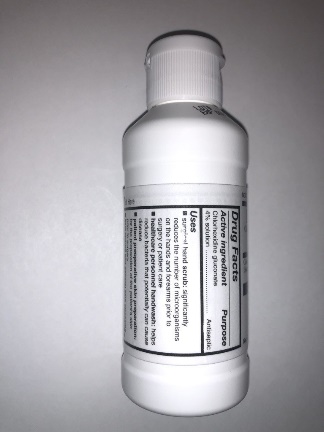 | **2% CHG Cloths**  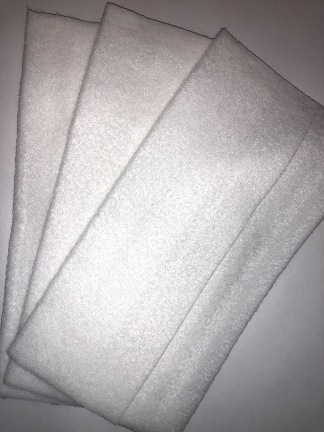 | **Soft Hair Ties**  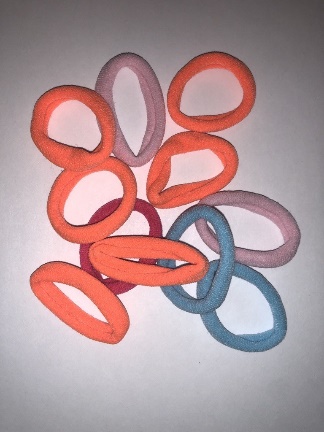 | **Wide-tooth Comb**  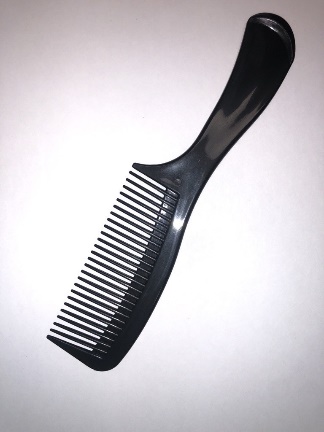 |

Supplemental Appendix 1**.** Protocol for post-craniotomy wound care providing directions to keep hair away from the incision,

daily cleansing and removal of incisional clots using CHG, and periodic CHG shampoo for hair. Cleansing occurred starting on post-operative day 1 or once the initial surgical dressing was removed per neurosurgeon instruction (generally by post-operative day 2). The protocol addresses aspects such as daily incision care, frequency of hair washing, and approved products.
